# The critical role of ferroptosis in virus-associated hematologic malignancies and its potential value in antiviral-antitumor therapy

**DOI:** 10.1080/21505594.2025.2497908

**Published:** 2025-04-29

**Authors:** Miao Miao, Yuelei Chen, Xuehan Wang, Shengyang Li, Rong Hu

**Affiliations:** a Department of Hematology, Shengjing Hospital of China Medical University, Shenyang, Liaoning, China; b College of Animal Science and Veterinary Medicine, Shenyang Agricultural University, Shenyang, Liaoning, China; c Shenyang Shenhua Institute Test Technology, Shenyang, Liaoning, China; d Publishing Department, Shenyang Agricultural University, Shenyang, Liaoning, China

**Keywords:** Hematological malignancies, EBV, KSHV, HTLV-1, ferroptosis, antiviral drugs

## Abstract

Epstein-Barr Virus (EBV), Kaposi’s sarcoma-associated herpesvirus (KSHV), and human T-cell leukemia virus type 1 (HTLV-1) are key infectious agents linked to the development of various hematological malignancies, including Hodgkin’s lymphoma, non-Hodgkin’s lymphoma, and adult T-cell leukemia/lymphoma. This review highlights the critical knowledge gaps in understanding the role of ferroptosis, a novel form of cell death, in virus-related tumors. We focus on how ferroptosis influences the host cell response to these viral infections, revealing groundbreaking mechanisms by which the three viruses differentially regulate core pathways of ferroptosis, such as iron homeostasis, lipid peroxidation, and antioxidant systems, thereby promoting malignant transformation of host cells. Additionally, we explore the potential of antiviral drugs and ferroptosis modulators in the treatment of virus-associated hematological malignancies.

## Introduction

As research on oncogenesis deepens, scholars have increasingly recognized the significant role played by viruses in tumor formation, with 16.1% of global tumors being linked to pathogenic agents [1]. One important commonality amo ng human oncogenic viruses is their ability to establish long-term, persistent infections in host cells without necessarily killing them. Upon invading host cells, viral genes regulate the physiological mechanisms of these cells, promoting cellular transformation and cancer development. The impact of viral infections on the hematological system is direct and evident, as viruses can act directly on the hematopoietic system or mediate hematological pathologies, including the onset of malignancies, through immune damage. Currently, only three viruses have been identified as causes of human hematological malignancies. The first is Epstein-Barr Virus (EBV), also known as Human Herpesvirus 4 (HHV4), a γ-herpesvirus initially discovered in endemic Burkitt’s lymphoma [2]. Subsequently, the pathogen responsible for adult T-cell leukemia was identified as HTLV-I [3]. The third virus, Kaposi’s Sarcoma-Associated Herpesvirus (KSHV, also known as HHV8), was found to be directly related to primary effusion lymphoma (PEL) [4]. Since the discovery of KSHV, no other viruses have been directly linked to human hematological malignancies, although viruses such as HIV and hepatitis C may contribute to hematological diseases through indirect mechanisms [5]. These viruses promote tumor initiation and progression through various mechanisms, posing a significant challenge to global public health. In this review, we will explore recent research advancements in hematological malignancies caused by the aforementioned viruses.

Cell death is an inevitable process that occurs naturally in all cells under both physiological and accidental conditions, and can be classified into accidental cell death (ACD) and regulated cell death (RCD) [6,7]. Cell death can be a double-edged sword; on one hand, virus-associated cell death helps prevent additional infections, while on the other hand, it can contribute to the progression of many infections [8,9]. To date, two main types of RCD have been identified in animal cells: apoptosis and non-apoptotic cell death. Although apoptosis has been extensively studied, there is increasing evidence that various forms of non-apoptotic RCD, such as pyroptosis, ferroptosis, necroptosis, cuproptosis, and autophagy-dependent cell death, are all related to pathogenic infections [10–13]. Ferroptosis is a relatively new form of regulated cell death that has become a hot topic in cancer and neurological diseases [14,15], yet its role in infectious diseases has been underestimated. Ferroptosis heavily relies on iron-mediated lipid radical formation and accumulation [16]. These processes can be inhibited by glutathione peroxidase 4 (GPX4) and the antioxidant glutathione (GSH). Ferroptosis is a possible outcome of pathogenic infections, with various pathogens manipulating it in different ways to promote pathogenesis or transmission [17]. Therefore, the discovery of ferroptosis provides new insights into the research and treatment of infectious diseases. Numerous studies have reported the connection between ferroptosis and viral infections.

Chemotherapy remains one of the primary treatment modalities for tumor diseases, whether they are caused by infectious or non-infectious factors. However, side effects and drug resistance have compelled researchers to explore more effective therapeutic approaches. In recent years, immunotherapy, targeted therapy, cell therapy, and hematopoietic stem cell transplantation have shown promising efficacy in various types of cancer. For viral infections that lead to tumor diseases, the value of antiviral drugs cannot be overlooked. By directly inhibiting viral replication and modulating host cell metabolism and immune responses to enhance therapeutic effects, antiviral drugs are often used in combination with chemotherapy [18,19].

This review aims to systematically summarize the relationships between Epstein – Barr virus (EBV), human T-cell leukemia virus type I (HTLV-I), Kaposi’s sarcoma-associated herpesvirus (KSHV) infections, and ferroptosis, exploring their roles in hematological malignancies. It also analyzes the potential clinical application value and challenges of targeting ferroptosis. The structure of this article is as follows: Firstly, it introduces the role of viral infections in hematological malignancies, with a focus on the oncogenic mechanisms of EBV, HTLV-I, and KSHV. Secondly, it delves into the molecular mechanisms of ferroptosis and how they are regulated by these viral infections. Finally, it discusses the prospects for the application of antiviral drugs and ferroptosis modulators in the treatment of virus-associated tumors, proposing future research directions. By systematically organizing these contents, this review hopes to provide valuable references for researchers in relevant fields, promoting the application of ferroptosis in the treatment of virus-associated tumors.

## Viral infection and hematological malignancies

In the realm of hematological malignancies, many human cancers are directly associated with infectious etiologies, with viruses being identified as the primary infectious agents. According to past research, EBV, HTLV-I, and KSHV play crucial roles in the onset of hematological malignancies [20–22]. These viruses are closely linked to the pathogenesis of malignant diseases such as Burkitt’s lymphoma, Hodgkin’s lymphoma, adult T-cell leukemia/lymphoma (ATL), and Kaposi’s sarcoma. This article will primarily introduce the latest research progress on hematological malignancies caused by these viruses.

### EBV and hematological malignancies

#### EBV

EBV, also known as Human Herpesvirus 4, is a γ herpesvirus with a double-stranded DNA molecule of 172,000 base pairs. EBV infection is highly prevalent globally, with over 90% of adults carrying the virus [23]. Nevertheless, EBV infection is typically subclinical, reflecting a symbiotic relationship between the virus and its host, where the virus needs to maintain the host’s health to sustain its own survival.

EBV primarily infects B lymphocytes and epithelial cells, establishing latent infections in B lymphocytes while undergoing viral replication in epithelial cells. T cells and NK cells are the main immune cells that control EBV infection, but they themselves can also be infected by EBV [24,25]. Consequently, in immunosuppressed individuals, EBV infection can lead to severe clinical outcomes [26]. Recent research has shown that EBV is a major contributor to the development of various diseases, particularly closely related to hematologic system disorders, including infectious mononucleosis [27], chronic active EBV infection [28], hemophagocytic lymphohistiocytosis [29], and various hematologic malignancies [30]. When the balance between the virus and the host immune system is disrupted, EBV can drive malignant transformation of lymphoid and epithelial origins, resulting in approximately 200,000 deaths annually [1,31].

EBV is indeed the first human tumor virus discovered that can transform quiescent B lymphocytes into lymphoblastoid cell lines (LCLs) in vitro [32]. The proteins encoded by the EBV genome primarily include Epstein-Barr nuclear antigens (EBNA1, EBNA2, EBNA3A, EBNA3B, EBNA3C, EBNA-LP), latent membrane proteins (LMP1, LMP2A, LMP2B), and two small RNAs associated with EBV (EBER1, EBER2) [33]. Regarding its oncogenic mechanisms, EBV promotes unlimited cell proliferation by encoding viral proteins such as Epstein-Barr nuclear antigens (EBNAs) and latent membrane proteins (LMPs) that interfere with host cell signaling. Additionally, one of the key mechanisms of EBV infection is to induce genomic instability leading to malignant transformation by affecting cell cycle regulation and DNA repair functions [34,35].

### Hodgkin lymphoma, HL

Hodgkin lymphoma (HL) is a malignant tumor that occurs in lymphoid tissues, characterized by the destruction of normal lymphoid tissue architecture and the presence of scattered characteristic mirror-image cells (Reed-Sternberg, RS cells) within a background of various infiltrating cells. The epidemiological and pathobiological mechanisms of HL are complex, and Epstein-Barr virus (EBV) is considered a major candidate in the pathogenesis of HL [36]. EBV latent membrane proteins (such as EBER, LMP1, and LMP2A) are commonly present in tumor cells of HL patients, and the correlation between EBV infection and HL is particularly evident in immunocompromised individuals [37].

The oncogenic mechanisms of EBV in HL involve multiple pathways. LMP1 and LMP2A are highly expressed in lymphoma cells, and these proteins enhance the survival and proliferation of tumor cells by mimicking the CD40 signaling pathway and B-cell receptor signaling. EBV induces immune evasion in host cells, weakening the immune system’s ability to attack tumor cells, allowing them to continue to proliferate [38]. In EBV-positive HL patients, there is a high presence of regulatory T cells (Treg) and Th2 cells, which secrete immunosuppressive cytokines such as IL-6, IL-8, and IL-10, further weakening the host’s anti-tumor immune response. The formation of an immunosuppressive microenvironment enables EBV-infected tumor cells to evade immune surveillance, increasing the tumor’s aggressiveness [39]. EBV-positive tumor patients have higher levels of vascular endothelial growth factor (VEGF) expression, leading to increased tumor angiogenesis, which is associated with poorer survival rates. By promoting tumor angiogenesis, EBV accelerates tumor growth and metastasis. Therefore, combining drugs targeting angiogenesis may have significant therapeutic value in the treatment of EBV-positive HL [40].

### Burkitt lymphoma, BL

Burkitt lymphoma (BL) is a highly aggressive mature B-cell tumor that includes three historically recognized subtypes: endemic BL, sporadic BL, and immunodeficiency-associated BL. EBV can be detected in over 90% of patients with endemic BL, while 5% to 15% of sporadic BL patients are associated with EBV infection, and 30% to 40% of immunodeficiency-associated BL patients are EBV-positive [41]. The oncogenic mechanisms of EBV in BL mainly involve its encoded latent membrane proteins and virus-encoded miRNAs. LMP-1 increases B-cell proliferation and inhibits apoptosis by mimicking the CD40 signaling pathway. The EBNA1 structure contains repeated glycine-alanine (Gly/Ala) sequences, which can impair protease action on EBNA1, resulting in antigen peptides that are difficult to bind to HLA class I molecules, thus failing to activate CD8 + T cells [42]. EBV-encoded miRNAs play important roles in BL, for example, miR-142 and BART-6-3 promote the proliferation and differentiation of lymphoma cells and regulate related immune responses by inhibiting PTEN expression, contributing to malignant transformation [43,44]. Additionally, c-MYC is a key oncogene in BL, and its overexpression in BL tumors is caused by IGH/c-MYC chromosomal translocation, leading to uncontrolled cell proliferation. Studies have shown that EBV may promote the occurrence of IGH/c-MYC translocation by inducing the expression of activation-induced cytidine deaminase (AID), thereby contributing to the development of BL [45]. Immunosuppression caused by malaria can increase the EBV load in the body. Meanwhile, the combined effect of malaria and EBV may activate AID, increasing the mutation rate of B cells and further increasing the risk of tumorigenesis [46]. There are still many unanswered questions regarding the interaction between EBV and BL. Although EBV is one of the main pathogenic factors of BL, not all individuals infected with EBV will develop BL. Therefore, studying the complex interactions between EBV and the host immune system, especially the influence of other factors such as malaria, will be a key direction for future research.

### Diffuse large B-cell lymphoma, DLBCL

Diffuse Large B-cell Lymphoma (DLBCL) is the most prevalent subtype of non-Hodgkin’s lymphoma, accounting for about 30% of all malignant lymphomas. The association between Epstein-Barr Virus (EBV) infection and DLBCL has attracted widespread attention, particularly in elderly patients and immunocompromised individuals, where EBV-positive DLBCL exhibits more aggressive features and a poorer prognosis. In a large-scale study conducted in Western countries, 4% of DLBCL cases were EBV-positive, with a median patient age of 60.5 years, and these tumors often showed activation of the NF-κB and JAK/STAT signaling pathways. Meanwhile, the incidence of EBV-positive DLBCL is higher in Asia and Latin America, accounting for about 15% of DLBCL cases, with this regional difference possibly attributed to genetic and environmental factors [47]. Patients with EBV-positive DLBCL typically present with an aggressive clinical course, including extranodal organ involvement (such as the skin, lungs, stomach, and central nervous system), and often accompany with B symptoms (such as fever, night sweats, and weight loss). Compared to EBV-negative DLBCL, patients with EBV-positive disease have a worse prognosis, especially with poor response to standard chemotherapy (such as the CHOP regimen) [48]. EBV infection promotes the development of DLBCL through various mechanisms. Tumor cells in patients with EBV-positive DLBCL usually express CD30, and there is high expression of immunosuppressive cytokines (such as IL-10) in their microenvironment, which inhibits the host’s anti-tumor immune response. Increased expression of PD-1 is also associated with poorer survival, suggesting that immune escape plays a crucial role in the development of EBV-positive DLBCL [47]. One of the pathogenic mechanisms related to EBV in DLBCL is immunosenescence. The weakening of the immune system makes it easier for EBV to evade immune surveillance, which explains the high incidence and poor prognosis of EBV-positive DLBCL in the elderly population [49].

### NK/T-cell lymphoma, ENKTL

NK/T-cell lymphoma (ENKTL), also known as extranodal natural killer/T-cell lymphoma, is an aggressive lymphoma closely associated with EBV infection. It is predominantly found in Asian and Latin American indigenous populations, but is rarely seen in native Europeans and North Americans [50]. ENKTL primarily occurs in extranodal sites, including the nasal cavity or paranasal sinuses, with fewer cases involving local lymph nodes. It is characterized by a vascular-centric and angiodestructive growth pattern mixed with apoptotic bodies forming coagulative necrosis. The viral products encoded by EBV contribute to the development of NK/T-cell lymphoma. For instance, LMP1 can stimulate the transcription of eukaryotic initiation factor 4E (eIf4E) through the NF-κB pathway, promoting the proliferation, migration, and invasion of EBV-infected NK/T cells [51]. LMP1 also increases the expression of PD-L1, thereby inhibiting the cytotoxicity of T-cells [52]. T-bet is a major transcription factor expressed in mature NK cells and T-lymphocytes. Lin et al. found that BART20-5p and miR-29, encoded by EBV, can inhibit the expression of the T-bet gene, reducing cellular immune function, and also inhibit the expression of P53 to promote tumor growth [53].

### Human T-cell lymphotropic virus type I (HTLV-I) and adult T-cell leukemia-lymphoma

HTLV-I is the first retrovirus discovered to be associated with human tumors, capable of causing adult T-cell leukemia-lymphoma (ATL) and tropical spastic paraparesis. The transmission routes of HTLV-I mainly include mother-to-child transmission, sexual contact, and blood transmission. Globally, over 10 million people are infected with HTLV-I, with approximately 3% potentially developing ATL, and the average latency period can be as long as 30 years [54]. The oncogenic process of the virus is complex, involving a combination of multiple factors such as host immune evasion, genomic instability, and chronic inflammatory responses. HTLV-I infects CD4 + T-cells, gradually leading to clonal expansion and malignant transformation of T-cells. Under the action of reverse transcriptase, HTLV-I synthesizes viral DNA, which integrates into the host cell and converts into different cell clones depending on the integration site. During continued cell proliferation, mutations occur in the cell clone’s DNA, evolving into leukemic cells and subsequently forming leukemic cell clones. Watanabe proposed [55] that the molecular basis of ATL involves up to five or more genetic events, particularly mutations in the T-cell receptor-NF-κB signaling pathway, such as PLCG1, PRKCB, and CARD11. These gain-of-function mutations drive clonal expansion and malignant transformation of cells. Additionally, the epigenetic characteristics of ATL are manifested as H3K27me3 accumulation, indicating that epigenetic changes are early events [55]. Kataoka et al.‘s comprehensive molecular analysis [56] revealed genetic and epigenetic alterations in ATL, including mutations in pathways related to T-cell signaling, cell migration, and immune surveillance. These genetic changes not only help elucidate the key molecules involved in T-cell malignant transformation but also provide potential targets for developing new diagnostic and therapeutic approaches.

HTLV-I infection not only causes clonal expansion of T-cells but also alters the immune system, enabling infected cells to evade host immune surveillance. A single-cell RNA sequencing study has revealed the immune activation status induced by HTLV-I infection and found that ATL cells exhibit a strong Treg phenotype, suggesting that these malignant T-cells may induce immune tolerance by expressing HLA class II molecules, thereby evading host immune surveillance [57]. The oncogenic proteins TAX and HBZ encoded by HTLV-I play decisive roles in the immortalization of T-cells and the abnormal regulation of host cell functions leading to leukemogenesis. TAX promotes cell cycle progression by deregulating oncogenic signaling pathways, playing a central role in the transformation of CD4 + T-lymphocytes. Additionally, TAX primarily targets the IκB kinase complex in the cytoplasm, inhibiting the autophagy pathway and resulting in sustained activation of NF-κB and upregulation of its responsive genes, which are crucial for T-cell survival and cell cycle progression. The Tax protein induces clonal proliferation of T-cells by promoting abnormal activation of the cell cycle and inhibiting apoptosis. Furthermore, Tax can integrate into the host genome, causing genomic instability and ultimately leading to malignant transformation of cells. The HBZ protein provides continuous stimulatory signals for the development of ATL by regulating cell proliferation and survival [58–60].

### Kaposi’s sarcoma-associated herpesvirus (KSHV) and hematologic malignancies

#### Kaposi’s sarcoma

The most typical and common manifestation of Kaposi’s Sarcoma-Associated Herpesvirus (KSHV) related to malignancy is Kaposi’s sarcoma. This is a tumor derived from vascular endothelial cells, which primarily manifests as lesions on the skin and mucous membranes but can also involve the lymphatic system, presenting as a hematological malignancy-like condition. Kaposi’s sarcoma is commonly observed in patients with compromised immune systems, particularly those with AIDS, and is therefore considered one of the common opportunistic infections associated with AIDS. KSHV is a linear double-stranded DNA virus with an icosahedral capsid, tegument, and envelope. Its genome contains approximately 90 open reading frames (ORFs) that encode proteins involved in viral replication, transcription, and protein synthesis. KSHV enters endothelial cells by binding to host cell surface receptors such as integrins, cystine-glutamate transporter xCT, heparan sulfate, and tyrosine protein kinase receptor EPHA2. The entry of KSHV leads to the viral capsid entering the cytoplasm, followed by uncoating and delivery of the KSHV genome into the nucleus. KSHV is capable of immune evasion to avoid attack by the immune system. K3 and K5 of KSHV are lytic genes encoding the modulators of immune recognition MIR1 and MIR2, respectively, both of which can inhibit antigen presentation by major histocompatibility complex (MHC) I to prevent the immune system from detecting KSHV-infected cells [61]. New research reveals that KSHV hijacks the host proteins CDK6 and CAD, causing infected cells to produce additional metabolites, thereby enabling faster viral replication and uncontrolled cell proliferation [62]. KSHV manipulates these two enzymes, CDK6 and CAD, to reshape the way human cells produce new nucleotides and process glucose. The growth pattern of infected cells and the persistence of KSHV are altered, significantly increasing the risk of cell tumorigenesis. Furthermore, KSHV interacts with Smad1, an important signaling molecule in the BMP pathway, through the LANA protein to promote the binding of Smad1 to the Id1 promoter, thereby upregulating Id1 expression at the transcriptional level [63]. This process may play a crucial role in promoting tumor cell proliferation and angiogenesis. Therefore, KSHV affects host cells through various mechanisms, including immune evasion, abnormal activation of signaling pathways, hijacking of host proteins, and alteration of cell metabolism, all of which collectively promote the onset and development of tumors.

### Primary effusion lymphoma, PEL

Primary effusion lymphoma (PEL) is a rare B-cell lymphoma characterized by the production of malignant effusion in the pleural and peritoneal cavities, closely associated with KSHV infection. PEL commonly occurs in patients with immunosuppression, particularly those with AIDS. It is also related to dual infection with KSHV and Epstein-Barr virus (EBV), with tumor cells often harboring both viruses. Unlike Kaposi’s sarcoma (KS), PEL consists of a monoclonal population of B cells, evidenced by clonal immunoglobulin gene rearrangements, with each tumor cell carrying a high copy number of KSHV. Morphologically, PEL exhibits features of immunoblastic and anaplastic large cell lymphoma [64]. Most PEL cells express CD45 and activation markers, including CD30, CD38, and CD7, as well as epithelial membrane antigen (EMA). However, PEL also expresses plasma cell markers such as CD138, VS38c, and MUM-1/IRF4, while the expression of B-cell-related antigens is relatively low. This suggests that although PEL originates from a monoclonal population of B cells, it is more inclined to be classified as partially differentiated plasma cells rather than mature B cells [65]. PEL is highly aggressive, and there is currently no standard treatment for it. The prognosis for patients with PEL is extremely poor, with an average survival time of approximately 6 months after diagnosis [66].

### Multicentric castleman disease, MCD

Multicentric Castleman Disease (MCD) is a lymphatic proliferative disorder closely associated with KSHV infection, characterized by the abnormal proliferation of IgM λ-restricted plasmablasts in the follicular mantle zone of B cells. MCD is more common in individuals with HIV infection, particularly in immunosuppressed hosts. It is often accompanied by systemic symptoms such as fever, weight loss, and hepatosplenomegaly, and in severe cases, it may lead to lymphoma-like lesions [67]. KSHV can replicate in plasmablasts within lymph nodes and transcribe a viral homolog of IL-6 (vIL-6), which induces VEGF production, promotes angiogenesis, and increases capillary permeability, leading to the symptoms, signs, lymph node pathological changes, and cytokine alterations associated with MCD [68]. vIL-6 stimulates B-cell proliferation, promotes excessive secretion of endogenous IL-6 (hIL-6) and increases NF-κB activity in patients, thereby inducing endothelial cells and inflammatory cells to secrete hIL-6, forming a positive feedback loop [69]. Studies have found that MCD patients have polymorphisms in the IL-6 receptor, with upregulated expression of soluble IL-6 receptor in the serum, and approximately 34% of patients respond to anti-IL-6 treatment, with improved IL-6 levels after treatment [70].

## The mechanism of ferroptosis

**Figure 1.**
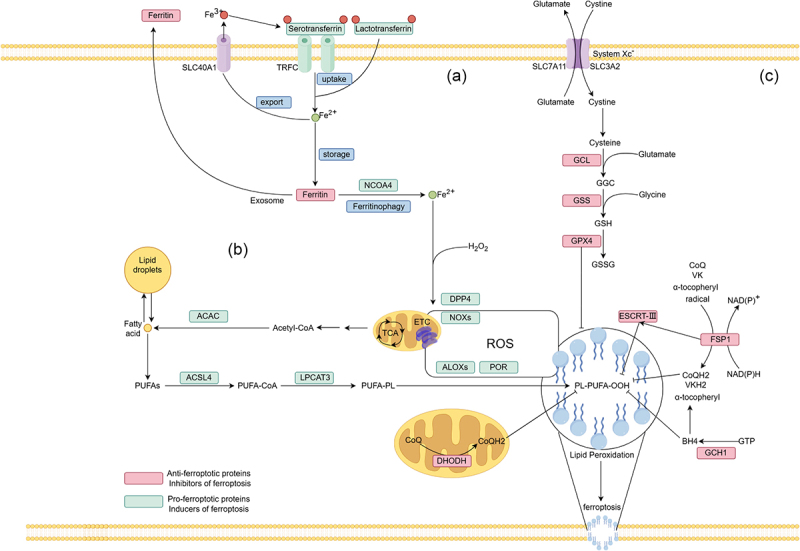
Main mechanisms of ferroptosis. (A) Iron homeostasis: transferrin and lactoferrin are capable of carrying iron ions and transporting them into cells via the transferrin receptor (TRFC). Ferritin, in conjunction with solute carrier family 40 member 1 (SLC40A1), facilitates the export of iron ions from the cell. These proteins, along with nuclear receptor coactivator 4 (NCOA4), control ferroptosis by regulating iron homeostasis. (B) lipid peroxide: fatty acid synthesis, mediated by acetyl-CoA carboxylase (ACAC), or the release of fatty acids from lipid droplets, leads to the formation of polyunsaturated fatty acids (PUFAs). These PUFAs are then integrated into phospholipids through the catalysis of long-chain acyl-CoA synthetase 4 (ACSL4) and lysophosphatidylcholine acyltransferase 3 (LPCAT3). Further, the generation of lipid peroxides is facilitated by lipoxygenases (ALOXs) through the production of reactive oxygen species (ROS), thereby promoting ferroptosis. (C) Cystine/glutamate transporter (system Xc−) system: the system xc- imports cystine into the cell while exporting glutamate. Intracellular cystine is reduced to cysteine, which is then used to synthesize glutathione under the catalysis of glutamate-cysteine ligase (GCL) and glutathione synthetase (GSS). GPX4 can utilize glutathione to reduce lipid peroxides to lipid alcohols. The antioxidant system centered around GPX4 effectively resists ferroptosis.

Ferroptosis is a form of cell death catalyzed by iron and dependent on reactive oxygen species (ROS) and lipid peroxidation. Unlike traditional forms of cell death such as apoptosis, necrosis, and autophagy, ferroptosis has a unique molecular mechanism centered on the accumulation of lipid peroxides and an imbalance in the cellular antioxidant system. The process of lipid peroxide accumulation within cells requires the involvement of iron ions, making ferroptosis iron-dependent. The main morphological characteristics of ferroptosis include increased mitochondrial membrane density, reduced mitochondrial volume, decreased or disappeared mitochondrial cristae, while the cell membrane remains intact and the nuclear morphology remains normal. The process of ferroptosis is regulated by a series of cellular metabolic pathways, including antioxidant regulatory systems, iron homeostasis, lipid overload, and other related signaling pathways (Figure 1). Focusing on the occurrence of ferroptosis in viral infections and elucidating the mechanisms by which viral infections utilize ferroptosis to promote disease progression will facilitate the development of new antiviral drugs.

### Iron homeostasis

Increased iron load is necessary for the accumulation of lipid peroxides and the occurrence of ferroptosis. The accumulation of Fe^2+^ in cells can increase the production of ROS through the Fenton reaction, leading to ferroptosis. Therefore, increased iron uptake, enhanced release, or restricted efflux of intracellular iron can all result in intracellular iron accumulation, triggering ferroptosis through various pathways. Circulating iron exists in the form of ferric ions (Fe3 + ) by binding to transferrin, and then Fe^3+^ enters the cell through the transferrin receptor 1 (TFR1). In endosomes, Fe^3+^ is reduced to ferrous ions (Fe^2+^) by ferrous reductase, and Fe^2+^ enters the labile iron pool in the cytoplasm from endosomes through the solute carrier family 11 member 2 (SLC11A2). Then, the solute carrier family 11 member 3 (SLC11A3) oxidizes Fe^2+^ to Fe^3+^ for excretion from the cell. Excess iron in normal cells is stored in ferritin, an iron storage protein complex that includes ferritin light chain (FTL) and ferritin heavy chain 1 (FTH1). Intracellular ferritin can be recognized by the specific receptor nuclear receptor coactivator 4 (NCOA4), which transfers ferritin to lysosomes for degradation after recognition, releasing iron ions and resulting in an increase in intracellular free iron [71]. Studies have shown that regulating iron metabolism through autophagy in cells can affect cell sensitivity to ferroptosis [72,73]. Therefore, the degradation of ferritin through lysosomes and autophagy pathways is considered a strong factor promoting ferroptosis [74]. Regulating iron metabolism and ferritin autophagy are potential key points for controlling ferroptosis.

### Lipid peroxidation

Lipids, as biomacromolecules containing hydrocarbons, serve as the structural basis for cell membranes or membrane-bound organelles (such as mitochondria, endoplasmic reticulum, and lysosomes). They are also key components for energy storage, insulation, intercellular communication, and signal transduction. Polyunsaturated fatty acids (PUFAs) are the main substrates in the lipid peroxidation stage during ferroptosis, especially arachidonic acid (AA), which is highly sensitive to peroxidation reactions. This sensitivity leads to the disruption of the lipid bilayer structure, subsequently impairing membrane function [75]. In contrast, monounsaturated fatty acids (MUFAs), due to their structural resistance to lipid peroxidation, have the function of inhibiting ferroptosis [76]. The production of PUFAs or MUFAs is regulated by various enzymes and is linked to various metabolic pathways. Acyl-CoA synthetase long-chain family member 4 (ACSL4) and lysophosphatidylcholine acyltransferase 3 (LPCAT3) are important driving enzymes for ferroptosis [77]. ACSL4 first catalyzes long-chain PUFAs, such as arachidonic acid and docosahexaenoic acid, to link them with coenzyme A (CoA). LPCAT3 then esterifies these acyl-CoA derivatives into phosphatidyl ethanolamines (PEs), which are subsequently oxidized by lipoxygenases (LOXs) into lipid hydroperoxides. These lipid hydroperoxides finally react with ROS to form toxic phospholipid hydroperoxides [78]. Knockout of ACSL4 and LPCAT3 can reduce the substrates for lipid peroxidation, thereby inhibiting the occurrence of ferroptosis [79,80]. However, providing exogenous AA or other PUFAs makes cells more sensitive to ferroptosis [81]. Conversely, the activation of MUFAs mediated by ACSL3 can replace PUFAs, inhibiting the accumulation of toxic lipid ROS and phospholipid levels, thereby suppressing ferroptosis [82,83]. Specific inhibitor-mediated inhibition of LOXs is an effective way to block ferroptosis, including 12/15LOX-ML351, 5LOX-zileuton, and tocopherols [84]. Additionally, some membrane electron transport proteins, such as NADPH oxidases (NOXs), contribute to the production of ROS, thereby promoting lipid peroxidation and ferroptosis [85].

### Antioxidant system

Intracellular iron accumulation and lipid peroxidation are the two central biochemical events in ferroptosis. Under normal circumstances, iron-mediated lipid peroxidation is strictly monitored by the intracellular antioxidant defense system. The phenomenon of ferroptosis was discovered during the screening of small molecule compounds for cancer treatment [86]. Currently, two small molecule activators of ferroptosis, erastin and RSL3, are known to be inhibitors of the antioxidant system, specifically inhibiting system Xc- and GPX4, respectively. GPX4 is an important antioxidant enzyme first identified in the process of ferroptosis, and its mechanism involves eliminating hydroperoxides in the lipid bilayer while preventing the accumulation of lethal lipid ROS [87]. As the first antioxidant system, System Xc- imports cystine into cells through a 1:1 glutamate antiport, and cystine is then converted into GSH through the glutathione metabolic pathway. GSH is a crucial member of the cellular antioxidant system and serves as an electron donor for GPX4. Together, they catalyze H2O2 and cytotoxic L-OOH into non-toxic lipid alcohols, thereby reducing oxidative stress damage [88]. System Xc- is composed of SLC7A11/xCT (solute carrier family 7 member 11) and SLC3A2 (solute carrier family 3 member 2), which are transporters for glutamate and cystine, facilitating their transport across the plasma membrane. Inhibition of System Xc- is one of the key factors in the activation of ferroptosis. A typical example is erastin, a widely used ferroptosis activator, which can impair the activity of System Xc- and thus trigger ferroptosis [89]. Currently, three additional mechanisms have been identified to be involved in the regulation of ferroptosis: the FSP1/CoQ10/NADPH pathway [90], the DHODH pathway [91], and the GCH1/BH4 pathway [92]. These pathways basically regulate intracellular reducing power such as GSH and CoQ10, and downregulate ROS levels to achieve the purpose of regulating lipid peroxidation and ferroptosis.

## Infection with EBV, HTLV-I, and KSHV and ferroptosis

HTLV-I, EBV, and KSHV are the three key viruses that cause hematological malignancies, each with distinct replication mechanisms. Elucidating the relationship between viral infection and ferroptosis is of great significance for the treatment of virus-associated hematological malignancies.

### EBV and ferroptosis

The products of EBV genes, such as LMP1, LMP2, EBV nuclear antigens, and EBV-encoded microRNAs, can induce imbalances in host lipid metabolism and redox homeostasis. This may affect the process of ferroptosis [93,94]. Recent studies have shown that inducing ferroptosis in EBV-associated tumors can effectively inhibit tumor growth and is an effective strategy to reduce resistance to radiotherapy and chemotherapy in EBV-related cancers [95,96].

EBV infection can influence tumor development and progression by regulating iron metabolism and oxidative stress levels. Research indicates that EBV-induced iron metabolism disorders, such as abnormal expression of hepcidin and ferritin, and enhanced oxidative stress, can promote ferroptosis, a process closely associated with EBV-related tumors like nasopharyngeal carcinoma (NPC) and Burkitt’s lymphoma [97–100]. The viral protein LMP1 can activate SREBP1, promoting the conversion of saturated fatty acids to monounsaturated fatty acids and reducing the content of polyunsaturated fatty acids, thereby inhibiting lipid peroxidation and decreasing cellular sensitivity to ferroptosis [101]. Additionally, LMP1 activates HIF-1α, which enhances the expression of SLC7A11, boosting the cell’s antioxidant capacity and thus suppressing ferroptosis [102]. Notably, EBV exhibits a dual regulatory mechanism on ferroptosis: it can induce ferroptosis by promoting iron accumulation and lipid peroxidation, but it can also inhibit ferroptosis by activating the p62-Keap1-NRF2 pathway, leading to increased expression of antioxidant factors such as SLC7A11 and GPX4, thereby maintaining viral survival [95]. Experimental evidence shows that inhibiting GPX4 significantly enhances the chemosensitivity of EBV-infected cancer cells, suggesting its potential role in mediating chemoresistance [95].

The study by Burton et al. [103] pointed out that he regulation of ferroptosis is dynamic. The EBV transformation program activates lipid metabolism, converting B cells into immortalized lymphoblastoid cell lines (LCLs). At different stages of EBV transformation, cells produce varying levels of lipid reactive oxygen species (ROS) byproducts, increasing the sensitivity of B cells to ferroptosis. However, during the growth phase of diseases similar to Burkitt’s lymphoma, GPX4 and its cofactor glutathione are required to counteract the accumulation of lipid ROS and avoid ferroptosis. In other words, the latency program of EBV dynamically affects the sensitivity of B cells to ferroptosis by regulating lipid metabolism and ROS production. Therefore, the bidirectional regulation of ferroptosis by EBV depends on the stage of infection, host cell type, and various factors in the microenvironment. This finding provides a new perspective for understanding the pathogenesis of EBV-related diseases and may contribute to the development of new therapies for EBV-related malignancies.

### HTLV-I and ferroptosis

In the research on the mechanisms of viral infection and ferroptosis, numerous studies have reported how different viral infections regulate intracellular iron metabolism to induce or inhibit ferroptosis. For instance, viral infections such as Hepatitis B (HBV) and Hepatitis C (HCV) can enhance or inhibit ferroptosis in host cells by modulating iron metabolism pathways. However, current research efforts have predominantly focused on inhibiting HTLV-1 viral replication or regulating immune responses, with relatively scarce studies on treating HTLV-1-related diseases through the regulation of ferroptosis pathways.

In other hematological malignancies, patients diagnosed with myelodysplastic syndromes (MDS) and acute myeloid leukemia (AML) often exhibit systemic iron overload, indicating an increased demand for iron by leukemic cells [104,105]. Although specific clinical studies on iron-related aspects in ATL (Adult T-cell Leukemia/Lymphoma) are currently lacking, studies have suggested that the gene expression profile of ATL is associated with susceptibility to ferroptosis. By analyzing the gene expression profiles of peripheral blood lymphocytes from ATL patients, 46 differentially expressed genes (DEGs) related to ferroptosis were identified. These genes are involved in pathways such as cellular response, chemical stress, and iron metabolism, and the functions of ferroptosis-related DEGs point to iron ion binding, transport, and uptake, indicating potential mechanisms of ferroptosis in HTLV-1-related diseases [106]. Furthermore, HTLV-1-infected T-cell lines abnormally express dihydroorotate dehydrogenase (DHODH) [107], whose activity is closely related to the cellular redox state and serves as one of the important defensive mechanisms against ferroptosis. DHODH supports NADPH production through the pyrimidine synthesis pathway, and NADPH is a crucial intracellular reductant that helps reduce GSH, maintaining an antioxidant state. When DHODH is inhibited, lipid oxidation and metabolic balance in cells are disrupted, particularly when pyrimidine synthesis is blocked, leading to oxidative stress and subsequently lipid peroxidation [91,108]. In recent years, DHODH inhibitors have shown potential in inducing ferroptosis, providing a new perspective for cancer treatment [109,110]. Therefore, the abnormal expression of DHODH caused by HTLV-1 infection is likely to be related to ferroptosis in some way.

Although the aforementioned studies suggest a potential association between HTLV-1 infection and ferroptosis, the direct molecular mechanisms remain unclear. Therefore, it remains to be determined whether HTLV-1 infection influences the fate of host cells through the regulation of ferroptosis pathways, and whether such regulation plays a crucial role in the pathogenesis of HTLV-1-related hematological tumors. Addressing these questions will provide new therapeutic approaches for HTLV-1-related cancers and other diseases.

### KSHV and ferroptosis

As an important regulator of ferroptosis, SLC7A11/xCT functions to import cystine for glutathione biosynthesis and antioxidant defense, and is overexpressed in various human cancers [111,112]. It has been reported that KSHV-encoded miR-K12-11 upregulates SLC7A11 to promote KSHV transmission and de novo infection. Targeting SLC7A11 induces apoptosis and tumor regression in KSHV/HIV-associated lymphomas [113–115]. High expression of SLC7A11 renders otherwise resistant target cells permissive to both KSHV cell fusion and viral particle entry [116,117]. These reports involving the function of SLC7A11 suggest that ferroptosis may be involved in KSHV infection and tumorigenesis.

A study has shown that Kaposi’s sarcoma-associated herpesvirus (KSHV) viral interleukin-6 (vIL-6) increases the activity of the deacetylase SIRT3, leading to the deacetylation of Serpine mRNA binding protein 1 (SERBP1). This deacetylation inhibits the stabilizing effect of SERBP1 on Lipid acyltransferase 2 (Lipt2) mRNA, subsequently causing Lipt2 degradation and inhibiting ferroptosis, thereby creating a favorable environment for cancer cell formation [118]. In addition, KSHV-transformed murine mesenchymal precursor cells (KMM) serve as a reliable model for studying KSHV-induced cell transformation. In KMM cells, there is a downregulation of ACSL4 and an upregulation of FTH1, while GPX4 expression remains unchanged [118]. This suggests that KSHV-induced ferroptosis inhibition may occur through the regulation of ACSL4 and FTH1 expression. Based on the above content, we can speculate that this ferroptosis inhibition is crucial for the tumorigenicity of KSHV. This interference mechanism of KSHV prevents cell self-destruction, allowing infected cells to survive under iron metabolic stress, thereby providing a conducive environment for tumor formation.

## EBV, HTLV-I, and KSHV in ferroptosis-related signal transduction

**Figure 2.**
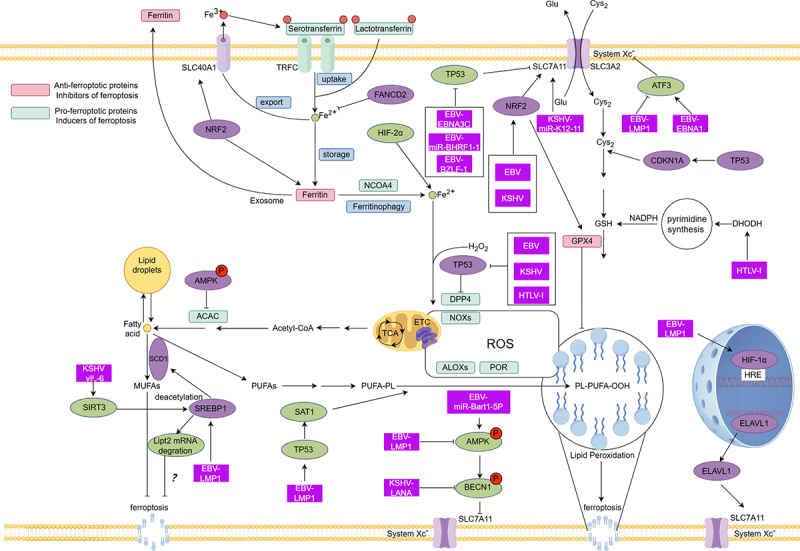
Ferroptosis related signaling pathway affected by EBV, HTLV-I and KSHV viruses. The oval represents key node proteins in the signaling pathway associated with ferroptosis. The square represents the virus or the gene product that the virus encodes. EBV, HTLV-I, and KSHV all inhibit TP53 (P53) signaling, while EBV and KSHV also activate NRF2. Specifically, KSHV’s viral interleukin-6 (vIL-6) enhances the activity of the deacetylase SIRT3, leading to the deacetylation of the serpine mRNA-binding protein 1 (SERBP1). This deacetylation inhibits SERBP1‘s ability to stabilize lipoyltransferase 2 (Lipt2) mRNA, resulting in the degradation of Lipt2 and the suppression of ferroptosis. However, the exact mechanism by which Lipt2 regulates ferroptosis remains unclear. NRF2, nuclear factor erythroid 2-related factor-2; HIF-1/2α, hypoxia-inducible factor alpha-subunit protein 1/2; AMPK, AMP-activated protein kinase; ATF3, activation transcription factor 3; SREBP1, sterol regulatory element-binding protein 1; SCD1, stearoyl-CoA desaturase; BECN1, beclin 1; ELAVL1, ELAV-like RNA binding protein 1; CDKN1A, cyclin-dependent kinase inhibitor 1A.

Current research highlights the involvement of multiple signaling pathways in ferroptosis regulation, including key pathways such as NRF2, P53, and AMPK-BECLIN1. Each pathway plays a distinct and critical role in ferroptosis by modulating iron metabolism, lipid metabolism, and oxidative stress. This section focuses on the mechanisms by which HTLV-I, EBV, and KSHV influence these ferroptosis-related pathways (Figure 2). Despite these mechanistic disparities, all three viruses converge on a common theme: reshaping ferroptosis susceptibility to favor viral persistence and oncogenesis.

### P53 signal

P53 is a core regulatory factor of tumor suppressor genes, responsible for cell cycle arrest, DNA repair, and the initiation of apoptosis. The mechanisms through which the P53 signaling pathway regulates ferroptosis are complex and multifaceted, with its effects potentially being either promoting or inhibiting under different physiological or pathological conditions. Specifically, P53 can directly bind to the P53 response element within the SLC7A11 promoter region, suppressing its transcription, which leads to a decrease in glutathione (GSH) synthesis and a reduction in GPX4 activity, ultimately triggering ferroptosis [119]. Additionally, when activated, P53 downregulates the expression of vitamin K epoxide reductase complex subunit 1-like 1 (VKORC1L1). By decreasing the expression of VKORC1L1, P53 reduces the production of reduced vitamin K (VKH2), a key antioxidant that inhibits ferroptosis. The reduction in VKH2 makes cells more susceptible to ferroptosis. Warfarin, an inhibitor of VKORC1L1, can enhance ferroptosis and inhibit tumor growth [120]. However,P53 also modulates lipid metabolism and redox genes, influencing the process of lipid peroxidation and increasing the likelihood of ferroptosis [121,122]. The impact of P53 on ferroptosis is context-dependent. In the presence of System Xc^−^ inhibitors (such as erastin), P53 preserves GSH and inhibits ferroptosis; whereas, in the presence of GPX4 inhibitors (such as RSL3), P53 promotes lipid peroxidation, thereby enhancing the sensitivity to ferroptosis. This difference may be related to cell cycle arrest [123].

EBV, HTLV-I, and KSHV all regulate the P53 pathway through different mechanisms. The mechanism by which EBV modulates P53 signaling is intricate. One of the EBV-encoded nuclear antigens, EBNA-3C, can form a complex with P53 [124]. This interaction may impact P53’s function, such as hindering its role as a transcription factor or altering its stability, thereby providing an environment favorable for viral survival and replication. Another significant discovery is that wild-type P53 can indirectly support EBV activity by stimulating the expression of LMP1 [125]. Specifically, when cells experience DNA damage, the elevation of P53 levels promotes an upregulation of LMP1, which in turn helps prevent apoptosis induced by DNA damage or mediated by interferon regulatory factor 5 (IRF5). This suggests a complex feedback regulation network during EBV infection. Given that MDM2 is one of the primary ubiquitin ligases responsible for P53 degradation, researchers have explored whether antagonizing MDM2 using small molecule inhibitors like Nutlin-3 could effectively suppress EBV-associated B-cell transformation and the growth of established lymphoblastoid cell lines [126]. The results showed that relieving MDM2-mediated inhibition of P53 indeed leads to apoptosis in these cells and inhibits viral transformation in newly infected cells.

Although the mechanisms behind HTLV-1-induced leukemia are not fully understood, the Tax protein encoded by HTLV-1 is considered a key factor in this process. Tax affects both normal and cancerous cellular processes by interfering with the synthesis or function of multiple regulatory factors, including the suppression of P53 tumor suppressor protein’s transcriptional activation. Studies indicate that MdmX, a transcriptional inhibitor of P53, is upregulated in HTLV-I-infected cells both in vitro and in vivo [127]. In most ATL patients, despite maintaining a wild-type status, P53’s function is inhibited. Tax prevents P53 from binding to the promoter regions of its target genes, thereby reducing the expression of important downstream effectors such as p21/CDKN1A [128]. This inhibition may be achieved through disrupting the function of histone acetyltransferase p300/CBP, leading to changes in chromatin structure that affect P53’s ability to recognize specific DNA sequences [10]. Further research has revealed that the NF-κB signaling pathway is involved in Tax-mediated P53 inhibition. Experiments have shown that treatment with specific NF-κB inhibitors can partially reverse the P53 dysfunction caused by Tax [129]. This suggests that NF-κB activation may be a crucial factor in the inactivation of P53 by Tax. Overall, through the complex regulation of P53 and related pathways, Tax not only promotes the malignant transformation of infected cells but also provides theoretical support for exploring new therapeutic strategies against ATL.

Research has shown that KSHV employs multiple mechanisms to modulate the function of P53, thereby promoting uncontrolled cell proliferation and tumor growth. One such mechanism involves the KSHV-encoded ORF45, which directly binds to P53, preventing its interaction with the deubiquitinase USP7 [130]. This interaction results in reduced P53 accumulation, cytoplasmic localization, and decreased transcriptional activity. Consequently, ORF45 functions as a novel inhibitor of P53 signaling and represents a potential therapeutic target for treating KS. Another study revealed that KSHV ORF50 inhibits P53-induced transcriptional activity and apoptosis by interacting with CREB-binding protein (CBP) [131]. This inhibition can be alleviated by increasing CBP levels, indicating the crucial role of CBP in this regulatory process. Additionally, vIRF-3, another KSHV-encoded protein, associates with the DNA-binding domain of P53, blocking its phosphorylation on serine residues and oligomerization [132]. vIRF-3 not only destabilizes P53 but also reduces its ability to activate downstream genes such as p21. Furthermore, vIRF1, encoded by KSHV, interacts with USP7, modulating its deubiquitination activity and thus inhibiting P53-mediated antiviral responses [133]. Moreover, KSHV upregulates Aurora A kinase levels, enhancing the interaction between P53 and the virus-encoded latent nuclear antigen (LANA), leading to increased P53 ubiquitination and degradation. This process is dependent on specific phosphorylation events at Ser215 and Ser315 [134]. Thus, KSHV uses a variety of strategies to influence the functional state of P53, including direct binding to P53 or its cofactors and altering the phosphorylation patterns of key signaling molecules. These mechanisms collectively contribute to weakening the host cell’s ability to combat viral infection and promote tumorigenesis.

The regulation of P53 by EBV, HTLV-I, and KSHV allows infected cells to evade clearance by the host immune system, particularly in a cancerous environment, where such regulation helps these cells survive and proliferate [135]. While EBV, HTLV-I, and KSHV use different mechanisms to control P53 signaling, their core objective is consistent: to dysregulate P53 function to facilitate persistent viral infection and oncogenesis. Although these mechanisms differ in their specific details, they all rely on direct or indirect interactions between viral proteins and host cell factors. This intricate regulatory network not only highlights the critical role of P53 in the development of blood cancers associated with viral infections but also provides a theoretical foundation for developing targeted antiviral and anticancer therapies.

### AMPK/BECLIN1 signal

AMP-activated protein kinase (AMPK) and Beclin-1 (BECN1) play crucial roles in the regulation of ferroptosis. AMPK, an energy-sensing protein, is involved in the modulation of cellular responses to energy stress and can activate autophagy [136]. The activation of AMPK not only induces ferroptosis through autophagy-dependent pathways but also promotes P53-mediated ferroptosis [136]. Additionally, AMPK directly inhibits the activity of system Xc- (a cystine-glutamate antiporter) by phosphorylating BECN1, thereby promoting ferroptosis [137]. Specifically, the phosphorylation of BECN1 at Ser90, Ser93, and Ser96 by AMPK is essential for the formation of the BECN1-SLC7A11 complex, which further suppresses system Xc- activity and induces lipid peroxidation [137]. BECN1, a key initiator of autophagy, controls the autophagic flux and intracellular transport processes as part of the PI3KC3 complex. Beyond its traditional role in autophagy regulation, BECN1 has been found to directly participate in the process of ferroptosis [138]. Moreover, the BECN1-SLC7A11 complex, formed independently of conventional autophagic pathways, provides an alternative mechanism for ferroptosis [139]. Thus, the AMPK/BECLIN1 signaling pathway integrates energy metabolism, autophagy, and lipid peroxidation, forming a multi-layered regulatory network for ferroptosis.

EBV-encoded miR-BART1 can directly target the host gene PTEN (phosphatase and tensin homolog), which is a negative regulator of the PI3K/AKT/mTOR signaling pathway. By suppressing PTEN expression, miR-BART1 leads to the activation of the AKT/mTOR signaling pathway, thereby inhibiting AMPK activity [140]. On the other hand, miR-BART1 can also regulate the AMPK/mTOR/HIF-1 pathway in a PTEN-independent manner by directly targeting the α1 catalytic subunit of AMPK (AMPKα1), promoting anomalous aerobic glycolysis and angiogenesis in nasopharyngeal carcinoma (NPC) cells [140]. EBV’s modulation of the AMPK signaling pathway via miR-BART1 reshapes host cell metabolism, balances autophagy and ferroptosis, evades immune surveillance, and facilitates tumorigenesis. This mechanism has been discussed in the context of EBV-associated NPC and is considered to offer a potential therapeutic strategy. This insight also holds significance for EBV-related hematological malignancies.

In current research, there are no definitive reports on the direct regulation of the AMPK-Beclin1 signaling pathway by HTLV-I. The key oncogenic proteins of HTLV-I, Tax and HBZ, have broad capabilities in modulating host signaling pathways, including NF-κB, mTOR, and autophagy [141,142]. In this context, HTLV-I may indirectly influence the AMPK-Beclin1 signaling to either activate or inhibit autophagy, thereby helping infected cells survive under conditions of energy depletion or high metabolic stress. 5-aminoimidazole-4-carboxamide riboside (AICAR), an AMPK activator, has been shown to induce apoptosis and mitochondrial membrane depolarization, leading to the death of ATL-related cell lines (S1T, MT-1, and MT-2) but not non-HTLV-1-infected Jurkat cells. However, this process does not increase AMPK phosphorylation levels [143], suggesting that AICAR’s therapeutic effect on ATL may be independent of AMPK. Resistance to ferroptosis via the AMPK-Beclin1 pathway, allowing for evasion of clearance, has been validated in EBV-associated tumors. Combining ferroptosis inducers, such as Erastin, with AMPK activators may selectively kill infected cells by blocking GPX4 function or increasing free iron pools, presenting a potential anticancer strategy. Although direct experimental evidence is lacking, modulating the AMPK-Beclin1 pathway may impact HTLV-I infection through multiple dimensions, including metabolic intervention, autophagy regulation, ferroptosis induction, and immune modulation, offering potential therapeutic value.

AMPK has also been identified as a key target in treating KSHV infections. Inhibition of endogenous AMPK activity increases viral yield by enhancing viral gene expression, while AMPK activators, such as AICAR and metformin, significantly suppress KSHV lytic replication by inhibiting the expression of viral lytic genes [144]. After 48 hours of KSHV infection in human umbilical vein endothelial cells (HUVECs), AMPK phosphorylation levels decrease, a result of PI3K/AKT/mTOR pathway activation [145]. At the molecular level, KSHV’s regulation of the AMPK-Beclin1 pathway may involve multiple mechanisms. The K1 protein encoded by KSHV binds to the AMPKγ subunit, affecting AMPK’s response to nutrient deprivation. This interaction promotes cell survival under stress conditions, enhancing viral persistence [146]. Additionally, the KSHV-encoded Bcl-2 homolog (cBcl-2) inhibits autophagy by binding to Beclin-1, blocking its role in the autophagic pathway. The virus utilizes this pathway to maintain latency and prevent cellular degradation [147].

In summary, activating the AMPK-Beclin1 signaling pathway has positive effects in limiting infections associated with EBV, HTLV-I, and KSHV. The use of AMPK activators, such as AICAR and metformin, alone or in combination with ferroptosis inducers, holds potential for treating blood malignancies related to these viruses.

### NRF2 signal

NRF2 (Nuclear factor erythroid 2-related factor 2) is an antioxidant stress regulator that activates a series of antioxidant genes, protecting cells from oxidative damage. KEAP1 (Kelch-like ECH-associated protein 1) acts as a negative regulator of NRF2, typically binding to it in the cell and promoting its degradation. NRF2 regulates ferroptosis through multiple mechanisms, including antioxidant defense, iron metabolism regulation, and control of the System Xc- pathway, which are crucial for maintaining cellular redox balance and inhibiting ferroptosis [148,149]. Some studies have shown that EBV can activate or maintain the NRF2 signaling pathway via LMP1 and KEAP1, enhancing the host’s antioxidant capacity and reducing oxidative damage caused by infection [95,150,151]. This mechanism not only aids in the long-term latency of EBV within the host but also supports cell proliferation and immune evasion, providing conditions for the development of EBV-related cancers [152].

In EBV-infected B cells, the virus-encoded latent membrane proteins LMP1 and 2A are key factors in promoting Nrf2 expression [150]. Research indicates that LMP1 and 2A facilitate the translocation of Nrf2 from the cytoplasm to the nucleus, activating downstream target genes and enhancing the antioxidant response, thereby supporting the proliferation of EBV-transformed B cells and increasing their resistance to chemotherapeutic drugs. The Akt signaling pathway plays a significant role in this process, with inhibition of Akt preventing Nrf2 activation [150]. In addition to the Akt pathway, MEK1/2 has been identified as a bridge connecting LMP1 to Nrf2. In T-cell lymphoma models, overexpression of LMP1 increases autophagy levels through the activation of the MEK1/2-Nrf2 axis, which helps resist the effects of anticancer drugs like cisplatin [153]. Furthermore, studies have found that EBV infection may upregulate GPX4 expression via the p62-Keap1-NRF2 signaling pathway, further decreasing sensitivity to ferroptosis and increasing resistance to chemotherapeutic agents [95]. Thus, EBV primarily modulates Nrf2 activity through LMP1 and associated signaling cascades, promoting cell survival, proliferation, and resistance to external stresses such as ferroptosis.

Research on the regulation of NRF2 by HTLV-1 is still in its early stages, with relatively few studies directly examining the interaction between HTLV-1 and NRF2. Tax, a key pathogenic protein of HTLV-1, interferes with normal cellular functions in various ways, such as activating the NF-κB signaling pathway, disrupting cell cycle control, inhibiting apoptosis, and inducing genomic instability [154,155]. Given the complex and nuanced relationship between the NF-κB and NRF2 signaling systems (which can either work synergistically to respond to stress stimuli or exhibit mutual inhibition due to resource competition) [156], the strong activation of NF-κB by Tax might indirectly affect the activity state or downstream effectors of NRF2. Another important protein encoded by HTLV-1, HBZ, also participates in the regulation of the NF-κB signaling pathway. Although there is currently no direct evidence that HTLV-1 targets NRF2 for regulation, based on the known modes of action of the virus and the critical role of NRF2 in cellular responses, it is likely that HTLV-1 indirectly alters the status of the NRF2 signaling network through interference with NF-κB signaling or other pathways. Future research may reveal more details in this area.

Compared to EBV and HTLV-1, the studies on KSHV-induced abnormalities in NRF2 function are more definitive. During primary infection of human dermal microvascular endothelial cells, KSHV rapidly activates Nrf2, primarily through a reactive oxygen species (ROS)-mediated mechanism, leading to the separation of Nrf2 from its inhibitory protein Keap1 and promoting Nrf2 phosphorylation and subsequent nuclear translocation [157]. During latent infection, KSHV continues to induce Nrf2 activation. Studies have shown that in long-term infected endothelial cells, KSHV achieves this by increasing the half-life of Nrf2, enhancing its phosphorylation by atypical protein kinase C (PKC-ζ), and promoting its nuclear localization [158]. Notably, at this stage, the activation of Nrf2 appears to be less dependent on an oxidative stress environment and more reliant on the action of the autophagy-related protein p62 [158]. The upregulation of Nrf2 during KSHV infection not only helps maintain the redox balance within host cells but also promotes a microenvironment favorable for viral survival and replication. Specifically, Nrf2 induces the expression of a series of antioxidant genes and other genes closely related to KS pathogenesis, such as COX-2 and VEGF-A [157]. Interestingly, these studies have also revealed interactions between Nrf2 and the KSHV latent protein LANA-1 and the host transcriptional repressor KAP1, collectively suppressing the expression of viral lytic genes [159]. Therefore, KSHV regulates Nrf2 activity through multiple pathways, promoting its own infection while also influencing the functional state of host cells. A deeper understanding of this complex molecular mechanism will help clarify the pathogenic mechanisms of other tumor-associated viruses, including EBV and HTLV-1, and may provide a theoretical basis for developing new treatments for blood system tumors related to viral infections.

## The crucial role of antiviral drugs in malignancies associated with EBV, HILT-1, and KSHV

Several antiviral drugs have been identified and are currently being evaluated for clinical use. These can be broadly classified into three categories: ① nucleoside analogs, such as Acyclovir (ACV), Ganciclovir (GCV), Penciclovir (PCV), and their oral prodrugs Valacyclovir (VACV), Valganciclovir (VGCV), and Famciclovir (FAM); ② nucleotide analogs, such as Cidofovir (CDV); ③ pyrophosphate analogs, including Foscarnet; and ④ immunomodulators, such as Interferon. The clinical efficacy of these antiviral drugs in viral infection-related malignancies requires further evaluation. Currently, antiviral therapy is the primary treatment for diseases related to KSHV infection, while for EBV and HILT-1-related malignancies, antiviral therapy is more commonly used as an adjuvant treatment to prevent further viral transmission.

### Antiviral drugs for EBV

The clinical efficacy of antiviral drugs in EBV-related malignancies remains unclear at present. To date, there is no effective antiviral therapy approved by the Food and Drug Administration (FDA) or the European Medicines Agency (EMA) for EBV infection. ACV and GCV effectively inhibit EBV in vitro. The effective dose of ACV for EBV (0.3 μM) is much lower than that for host cells (250 μM) [160], thus exhibiting a very favorable therapeutic index. Antiviral drugs (e.g. ACV, GCV, or VACV) have been shown to significantly reduce the incidence of post-transplant lymphoproliferative disorder (PTLD) in high-risk EBV-seronegative lung transplant patients [161] and decrease EBV viremia in pediatric renal transplant patients [162]. CDV possesses both antiviral and antiproliferative properties and can be metabolized into its active form by cellular kinases [163]. Studies have shown that intratumoral injection of CDV inhibits tumor growth in EBV-positive nasopharyngeal carcinoma xenografts in nude mice [164]. Another study demonstrated that treating nasopharyngeal carcinoma and lymphoma cell lines with cidofovir reduces the expression of LMP1 and EBNA2 oncoproteins and increases apoptosis [165]. Foscarnet, a pyrophosphate analog, exhibits broad antiviral activity against herpesviruses [166] by inhibiting the cleavage of pyrophosphate from nucleoside triphosphates, disrupting viral DNA polymerase activity. Unlike ACV and GCV, the activity of foscarnet does not depend on viral protein kinases, making it a potential option in cases of acquired resistance to ACV/GCV. However, patient tolerance may be low due to increased toxicity [167]. Perrine et al. [168] found through clinical trials that the combination of arginine butyrate and GCV is effective in treating EBV-related lymphomas. Arginine butyrate activates the EBV thymidine kinase (EBV-tk) encoded by bxlf1 in EBV-infected cells, making them sensitive to antiviral drugs such as GCV. This provides us with an idea that EBV proteins regulate many signaling pathways, and antitumor drugs inevitably affect normal signaling pathways or molecules, leading to serious side effects. Therefore, the multi-target regulatory effects of multiple drugs will play a key role in enhancing the efficacy of antiviral therapy.

### Antiviral drugs for KSHV

Antiviral drugs are the primary method for treating KSHV infections and related diseases. It is known that a small number of cells displaying lytic replication play a central role in the oncogenesis of Kaposi’s sarcoma (KS) [169]. However, compared to KS, a higher proportion of infected cells expressing lytic genes are found in primary effusion lymphoma (PEL) and multicentric Castleman’s disease (MCD) (up to 25% in MCD). Therefore, antiviral drugs may be more effective in treating MCD and PEL than in treating KS. Currently, the only “direct-acting antiviral drug” available for KSHV is the KSHV DNA polymerase inhibitor. Drugs effective against KSHV also include ganciclovir (GCV), cidofovir (CDV), and others [170]. Among them, GCV is a relatively important clinical medication in the treatment of KSHV infections. Compared to GCV, other drugs such as acyclovir (ACV), penciclovir (PCV), foscarnet (H2G), and A-5021 show weaker or no anti-KSHV activity. GCV, a DNA polymerase inhibitor, upon entering KSHV-positive tumor cells, is phosphorylated into monophosphate by a virus-encoded protein kinase homolog, and subsequently phosphorylated into diphosphate and triphosphate, inhibiting DNA synthesis. Numerous studies have demonstrated that GCV can reduce KSHV shedding in the saliva of KSHV-infected individuals [171]. Moreover, The GCV loaded within the nanodelivery system significantly inhibited viral lytic replication in vivo, thereby exerting an anti-KSHV effect [172]. Both GCV and CDV have been reported to successfully treat PEL when used alone or in combination with adjuvant chemotherapy, immunotherapy, or highly active antiretroviral therapy (HAART) [173–175]. Furthermore, some MCD patients have been successfully treated with GCV [176,177], while CDV has failed [178]. Due to the complexity of the physiological environment in vivo, the non-specific effects and toxic side effects of GCV severely limit its therapeutic efficacy, and resistance can easily develop. The efficacy of GCV against KSHV still needs further optimization.

### Antiviral drugs for HTLV-1

ATL is an extremely difficult-to-treat hematological disease. Despite advancements in technology, people have gained a deep understanding of the onset and progression of ATL, and chemotherapy and other treatment modalities have been widely used in the clinical treatment of ATL. First-line combination chemotherapy drugs for ATL include VCAP-AMP-VECP (VCAP: vincristine, cyclophosphamide, doxorubicin, prednisone; AMP: doxorubicin, lomustine, prednisone; VECP: vincristine, etoposide, carboplatin, prednisone), etc [179,180]. However, regardless of the chemotherapeutic drugs chosen, the results are often unsatisfactory. To improve the cure rate of ATL, it is particularly important to seek more effective treatment methods.

Antiviral therapy has garnered widespread attention and is also controversial. In tissue culture, several antiretroviral drugs used for HIV treatment have been shown to be effective against HTLV-1, including the reverse transcriptase inhibitors zidovudine (ZDV) and tenofovir, as well as the integrase inhibitor raltegravir [2]. ZDV binds to the viral DNA polymerase, terminating the growth of the DNA chain and thereby inhibiting viral replication. It is primarily used for the treatment of AIDS or AIDS-related syndromes and for the treatment of immune deficiency virus (HIV) infections. Immunomodulators, such as interferons, enhance the host’s resistance to viruses by regulating the immune system. Numerous studies have demonstrated that the combination of interferon-α (IFN-α) and ZDV is highly effective in the treatment of ATL, with a high response rate in previously untreated ATL patients [181]. The combination of IFN-α and ZDV further induces P53 signaling and apoptosis in cells [182]. This approach is mainly used for patients with acute, chronic, and smoldering ATL, but has poor therapeutic effects on patients with lymphoma-type ATL [183]. A trial using EPOCH chemotherapy combined with bortezomib to block NF-κB activation and raltegravir as an antiviral drug for the treatment of acute ATL and ATL lymphoma showed good tolerability and a response rate of 67% [184]. Therefore, there is increasing evidence that controlling viral spread is a crucial aspect of ATL treatment.

Some studies have attempted to enhance the efficacy of antiviral therapy through drug combinations. For example, the addition of AS_2_O_3_ to ZDV and IFN-α may have excellent therapeutic effects on ATL by inducing activity targeted at leukemia-initiating cells [185]. Based on this, observations have shown that the combination therapy of AS_2_O_3_, IFN-α, and ZDV achieved a 100% response rate in 10 patients with chronic ATL [186]. This triplet combination appears to play a crucial role in restoring immune responses by transforming the ATL microenvironment from an immunodeficient state before treatment to an immunocompetent state. Although these studies are preliminary, the results suggest that the combination of AS_2_O_3_ with antiviral therapy can effectively inhibit the progression of ATL and is one of the promising targeted therapies for ATL [187]. Furthermore, AS_2_O_3_ triggers preventive and therapeutic immune responses against solid tumors by inducing necroptosis and ferroptosis [188]. Therefore, exploring the use of ferroptosis regulators to enhance the efficacy of antiviral therapy for ATL through the ferroptosis pathway is also a promising research direction.

## Future directions

RCD has been identified as a host defense strategy to restrict viruses, yet the mechanisms by which RCD influences viral pathogenesis are unique and limited. Ferroptosis, a type of RCD that has garnered significant attention in recent years, may play a complex but crucial role in viral infections. Compared to RCD in other pathogenic infections, research on the ferroptosis-pathogen interaction remains insufficient. Furthermore, some areas of the mechanisms involved in virus-regulated ferroptosis are still unexplored, and specific pathogen infections such as EBV, KSHV, and HTLV-1 require further investigation to provide more details for the creation of new therapeutic strategies in antipathogenic drugs or vaccines. Most viruses utilize ferroptosis to promote their replication and release, which may facilitate viral dissemination and lead to host organ damage. We need to consider how to regulate the extent of ferroptosis to help the host eliminate pathogens while minimizing the physiological harm to the host caused by ferroptosis. Currently, research on ferroptosis and viral infectious diseases is in its infancy. Exploring the interaction between ferroptosis and infectious diseases and proposing effective treatment methods hold significant value and represent a future direction for research on the treatment of infectious diseases.

The pathogenesis of hematologic malignancies is complex, often involving multiple pathways and targets. Traditional multi-drug combination chemotherapy with cytotoxic agents can easily lead to serious adverse consequences such as bone marrow suppression and immune disruption. The aforementioned review highlighted the critical application value of antiviral drugs in virus-associated malignancies. However, antiviral drugs face challenges in the treatment of EBV, KSHV, and HTLV-1-related malignancies, including limited efficacy, a lack of drug variety, and side effects. Although novel therapies such as interferons and monoclonal antibodies show promise, more research is needed to confirm their efficacy. Future developments in the use of antiviral drugs for EBV, KSHV, and HTLV-1-related malignancies will mainly focus on the development of new drugs, multi-target antibody combination therapies, and in-depth research into viral gene mechanisms [189–192]. These research directions are expected to provide new strategies and methods for the treatment of virus-associated malignancies. Additionally, regulating iron may help reduce the adverse consequences of traditional chemotherapy in infectious hematologic malignancies. Tumors associated with viral infections typically exhibit complex metabolic reprogramming and drug resistance characteristics. Ferroptosis regulators, by targeting iron metabolism, lipid peroxidation, and antioxidant systems, can provide novel therapeutic targets for these refractory tumors, potentially overcoming the limitations of conventional treatments. Desferioxamine, an iron chelator, is widely used to treat iron overload. Studies have reported that Desferioxamine can induce the EBV lytic cycle in some cancer cells by inhibiting the enzymatic activity of protein hydroxylases [193]. A new compound called C7 has recently been described and reported to trigger the ERK-autophagy axis to reactivate the EBV lytic cycle through intracellular iron chelation [194], and drug-induced activation of latent EBV into the lytic cycle in tumor cells is one of the potential targeted therapeutic strategies for EBV [195]. However, due to the abundance of iron in the in vivo microenvironment, delivering iron chelators to tumors remains challenging. Moreover, certain ferroptosis inducers, such as RSL3, exhibit significant toxicity, necessitating the development of targeted delivery systems to minimize collateral damage to healthy tissues [196]. Additionally, ferroptosis regulators have the potential to induce oxidative stress, highlighting the need for a thorough assessment of their cytotoxic effects on normal cells. While targeting ferroptosis offers a promising strategy to surmount resistance to conventional cancer therapies, tumor cells may also evolve resistance to ferroptosis regulators through alternative mechanisms. For instance, cancer cells can evade ferroptosis by upregulating antioxidant enzymes like Nrf2 or by reprogramming lipid metabolism. Therefore, a deeper understanding of these resistance mechanisms is essential, and the development of combination therapies that can effectively counteract such resistance is a critical next step in advancing ferroptosis-based cancer treatments. Existing research suggests that compounds that promote ferroptosis through iron metabolism, lipid metabolism, and glutathione metabolism can play a role in the field of oncology. Further exploration of drugs that induce ferroptosis in infected cells is needed, and high-throughput functional screening and AI-based methods may accelerate the development of ferroptosis-targeting drugs. Ferroptosis research holds promise for developing innovative antiviral and anticancer therapies by selectively inducing the death of infected or malignant cells through targeted modulation of iron-dependent cell death mechanisms.

## Data Availability

Data sharing is not applicable to this article as no new data were created or analyzed in this study.
